# Dosimetric superiority of flattening filter free beams for single-fraction stereotactic radiosurgery in single brain metastasis

**DOI:** 10.18632/oncotarget.13085

**Published:** 2016-11-03

**Authors:** Youqun Lai, Shanyu Chen, Changdong Xu, Liwan Shi, Lirong Fu, Huiming Ha, Qin Lin, Zhen Zhang

**Affiliations:** ^1^ Department of Radiation Oncology, The First Affiliated Hospital of Xiamen University, Xiamen, PR China

**Keywords:** flattenig filter free, volumetric modulated arc therapy, stereotactic radiosurgery, brain metastases

## Abstract

For single-fraction stereotactic radiosurgery (SRS) using linac in brain metastases, more accurate treatment delivery with higher tumor absorbed doses and lower absorbed doses to normal tissues remains an enormous challenge. The purpose of this study was to investigate the dosimetric superiority in flattening filter free beams (FFF) for volumetric modulated arc therapy (VMAT) in single brain metastasis. 68 patients with single brain metastasis were included in this study. Every patient was subjected to VMAT treatment plans using 6 MV standard flattened (FF) beams (VMAT_FF) and 6 MV FFF beams (VMAT_FFF) with single fraction doses of 20 Gy. Dosimetric evaluation was performed by analysis of target coverage, dose gradients, beam-on time (BOT), gantry speed and number of monitor units (MU). There were no differences between VMAT_FF and VMAT_FFF plans in conformity and MU. VMAT_FFF plans showed obvious superiority in homogeneity, dose gradients and efficiency. For the mean BOT, VMAT_FFF plans provided a significant decrease by 42.8% compared with VMAT_FF. By the use of FFF beams, brain irradiation was minimized with about 2% reductions in low-dose regions (about 5-10 Gy). FFF beams not only resulted in more efficiency by reducing treatment time, but also provided further brain sparing compared to traditional techniques for SRS in single brain metastasis.

## INTRODUCTION

Brain metastases are the common tumor that has spread to the brain from another cancer in the body, such as lung cancer, breast cancer and genitourinary tract cancers [1–3]. In the past few decades, surgery, radiation therapy and chemotherapy have become more effective treatments, especially stereotactic radiosurgery (SRS) is being increasingly utilized for the treatment of brain metastases [4–6].

There are many approaches for the treatment of brain metastases with SRS technology, such as Gamma Knife [7], CyberKnife [8], helical tomotherapy [9] and linear accelerator [10–12]. With more and more widespread and profound application of linear accelerator in radiotherapy, particularly the appearance of flattening filter free (FFF) beams in recent years, several investigators have studied the role of FFF beams for SRS in brain metastases [13–14]. The use of volumetric modulated arc therapy (VMAT) for SRS with FFF beams has recently been shown to shorten treatment time if compared with traditional flattening filter (FF) beams [15–16]. FFF beams potentially increase dose rates substantially shorten beam-on time (BOT). This is an important issue in radiation therapy, especially with single doses up to 20 Gy. Furthermore, a lower peripheral dose is its unique characteristic in FFF beams due to the decrease of photon head scatter, head leakage and leaf transmission [17]. However, several investigators have assessed the dosimetric comparison between FFF beams and FF beams, and the results showed that dose distributions achieved with FFF beams are similar to those with FF beams [16, 18]. J. Rieber, et al. [19] have compared plan quality of radiosurgery in brain metastases using 3-dimensional conventional technique, and showed that FFF beams provides similar plan quality with slightly reduced dose spillage to normal brain.

FFF beams resulted in a time efficient treatment delivery, especially when used in combination with VMAT technology. In this planning study, we evaluated the dosimetric superiority in FFF beams for single-fraction SRS in single brain metastasis by use of VMAT technology. Since the tumor in brain metastases is usually spherical, in order to keep the dose to healthy brain tissue below acceptable thresholds, the dose gradient has to be very steep [20]. We analyzed our 68 patients treated in our department with this technique, and focus on the dose gradient advantage using FFF beams compared with FF beams.

## RESULTS

### PTV coverage and dose distribution

The dosimetric parameters of PTV for treatment plans using FF and FFF beams were presented in Table 1. The value of D_90%_ was 20 Gy in both of these two treatment techniques because all plans were normalized so that 90% of PTV received 100% of the prescribed dose. There was no difference between VMAT_FF and VMAT_FFF plans in conformity. For the homogeneity, the values of D_max_ and V_110%_ were reduced by approximate 0.2 Gy and 3.7% respectively in regard to the values of D_max_ and V_110%_ by the use of VMAT_FFF techniques. Figure 2 showed the dose distributions of two planning techniques for one patient with single brain metastasis in axial, coronal, and sagittal views. The 2% reductions in dose exposure may not seem any difference with the naked eye in dose distributions. The DVH comparison for the PTV and brain with these two treatment plans was displayed in Figure 3.

**Table 1:** Dosimetric parameters of PTV and healthy brain tissue for treatment plans using FF and FFF beams

PTV Volume (cm^3^) =5.35 ± 3.88, range (cm^3^) = (0.4-14.1)			
	VMAT_FF	VMAT_FFF	*P* value
D_90%_ (Gy)	20 ± 0	20 ± 0	
D_mean_ (Gy)	21.2 ± 0.2	21.1 ± 0.1	<0.001
D_2%_ (Gy)	22.3 ± 0.2	22.2 ±0.2	<0.001
D_max_ (Gy)	22.8 ± 0.2 (22.2-23.4)	22.6 ± 0.2 (22.2-23.2)	<0.001
V_110%_ (%)	10.6 ± 7.1 (0.15-29.84)	6.9 ± 6.2 (0.01-25.1)	<0.001
CI	1.14± 0.06 (1.08-1.44)	1.14 ± 0.06 (1.08-1.48)	
HI	1.13 ± 0.01 (1.09-1.15)	1.12 ± 0.01 (1.09-1.14)	<0.001
GI_High_	2.893 ± 0.56 (2.274-5.124)	2.838 ± 0.54 (2.228-4.985)	<0.001
GI_Low_	2.732 ± 0.23 (2.289-3.497)	2.725 ± 0.22 (2.285-3.421)	0.027
V_50%Presc.Dose_ (cm^3^)	17.5 ± 9.84 (3.5-37.86)	17.19 ± 9.7 (3.42-37.5)	<0.001
V_25%Presc.Dose_ (cm^3^)	46 ± 23.36 (11-98.19)	45.19 ± 23.19 (10.58-97.53)	<0.001
**Brain** **Volume (cm^3^) =1344.7 ± 141.1, range (cm^3^) = (1038.9-1676.3)**			
D_mean_ (Gy)	0.941 ± 0.46	0.935 ± 0.46	<0.001

*Abbreviations*: VMAT_FF: VMAT plans using conventional flattened (FF) beams; VMAT_FFF: VMAT plans using flattening filter free (FFF) beams; CI: conformity index; HI: homogeneity index; GI_High_ = V_50%Presc.Dose_/V_90%Prescr.Dose_; GI_Low_ = V_25%Presc.Dose_/V_50%Prescr.Dose_; V_50%Presc.Dose_ is the volume of the body that received 50% of the prescribed dose. V_25%Presc.Dose_ is the volume of the body that received 25% of the prescribed dose.

**Figure 1:**
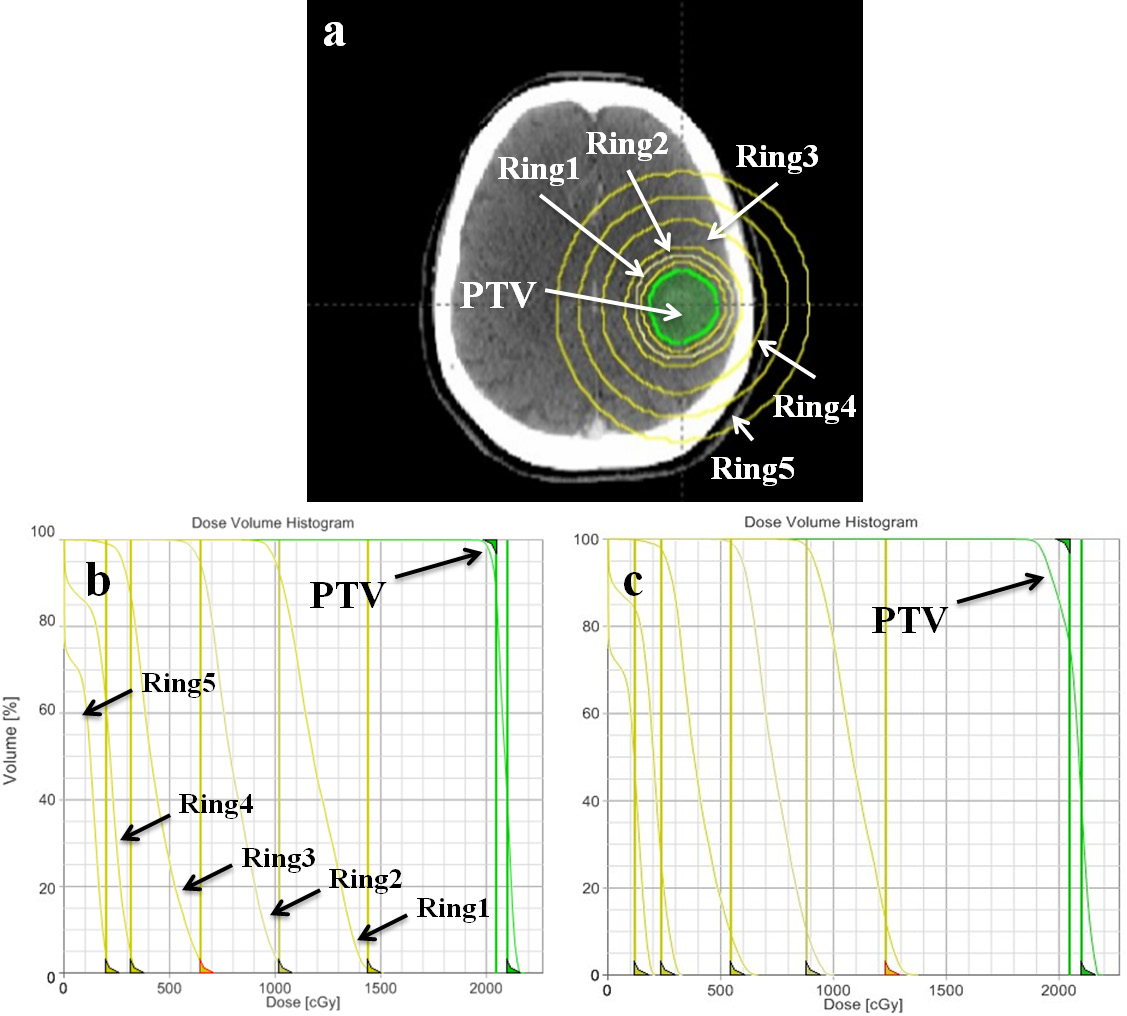
**a**. Delineated planning target volume and help structures (five rings) in single brain metastasis for optimization. Dose-volume histogram (DVH) of PTV and help structures in the first **b**. and second **c**. stage of optimization process.

**Figure 2:**
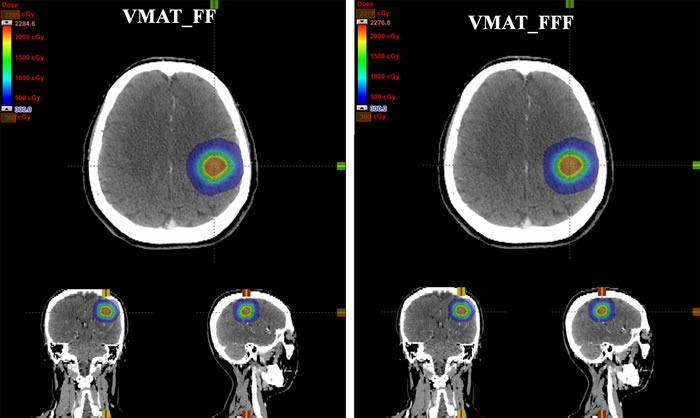
Dose distributions for one patient with single brain metastasis in axial, coronal, and sagittal planes using two different treatment planning techniques: VMAT_FF = VMAT plans with conventional flattened (FF) beams; VMAT_FFF = VMAT plans with flattening filter free (FFF) beams

**Figure 3:**
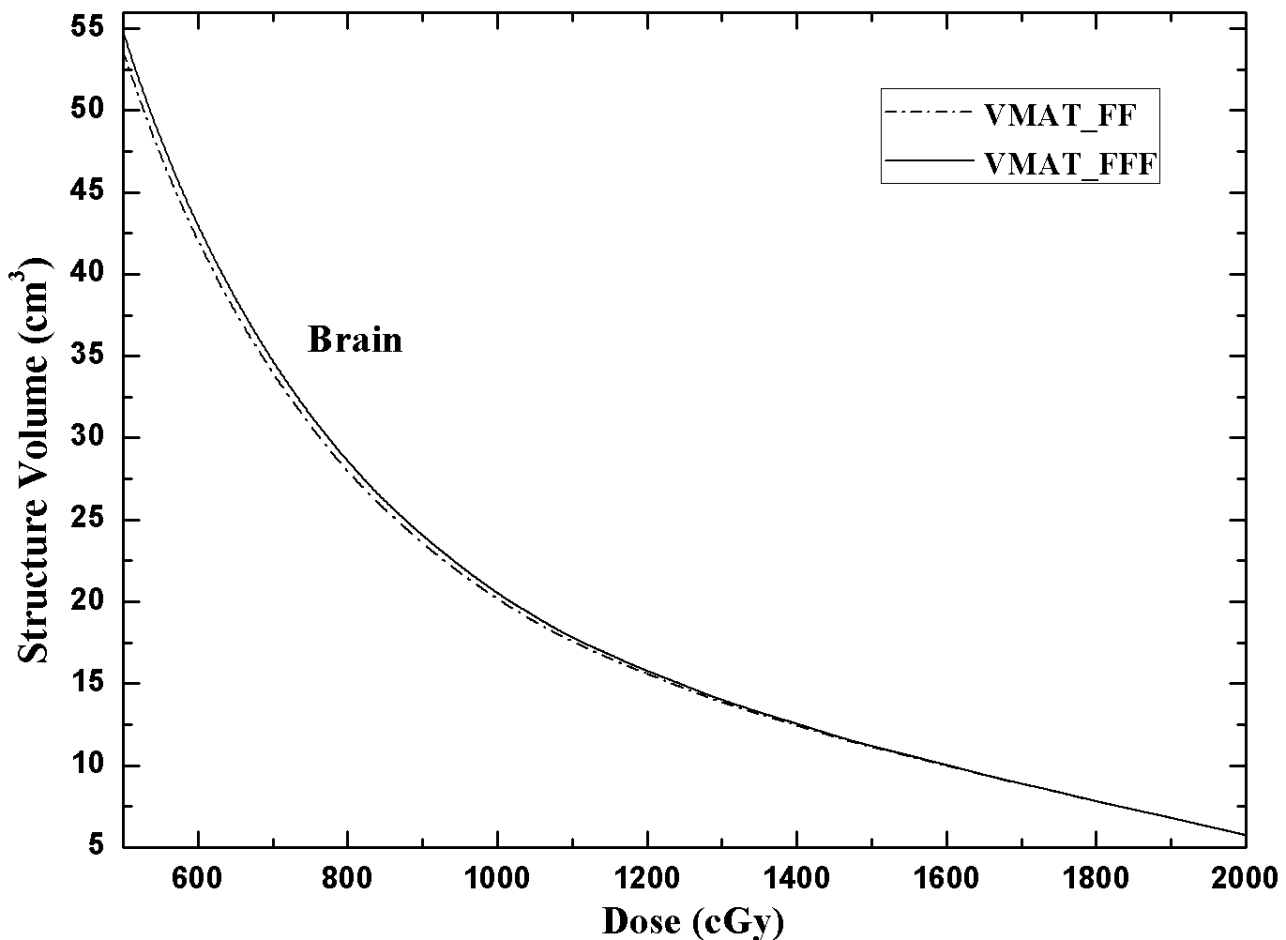
DVH comparison for the brain of the two different treatment planning techniques: VMAT_FF = VMAT plans with FF beams; VMAT_FFF = VMAT plans with FFF beams

### Dose to organs at risk

For brain irradiation, Figure 3 showed the DVH comparison for brain with two treatment plans. VMAT_FFF technique was slightly superior in dose exposure to healthy brain tissue when compared to VMAT_FF plan. With respect to the values of V_50%Presc.Dose_ (10 Gy) and V_25%Presc.Dose_ (5 Gy) in body, the VMAT_FFF plans showed a reduction of approximate 0.31 cm^3^ and 0.81 cm^3^ in the respective mean volumes (Table 1). The mean dose to healthy brain tissue was very small since the maximum diameter of tumor was smaller than 3 cm.

### Dose gradient

**Figure 4:**
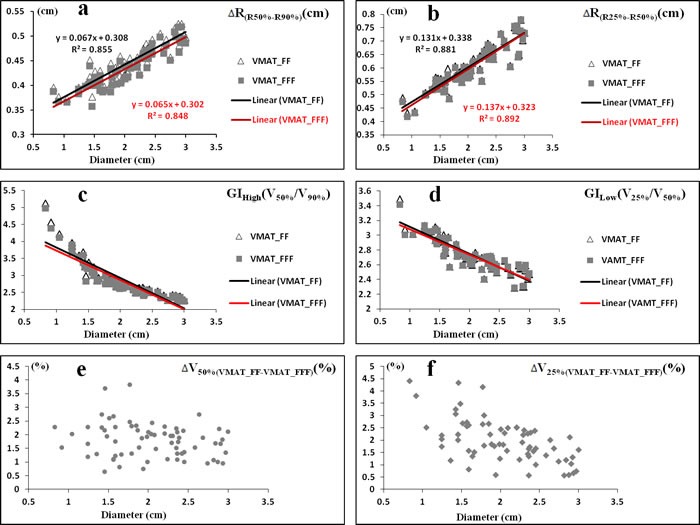
The linearity of the difference in ΔR (R50%-R90%). **a**., ΔR_(R25%-R50%)_ **b**., GI_High_ **c**. and GI_Low_ **d**. on the diameter of PTV using FF beams (Δ) and FFF beams (■) for all patients. The scatter plot of all patients in ΔV_50%(VMAT_FF-VMAT_FFF)_ **e**. and ΔV_25%(VMAT_FF-VMAT_FFF)_ **f**. ΔR_(R50%-R90%)_ = the increment of semidiameter between R50% and R90% (R_X(%)_ is equivalent sphere semidiameter for the volume received X% of the prescribed dose); ΔR_(R25%-R50%)_ = the increment of semidiameter between R25% and R50%; ΔV_X%(VMAT_FF-VMAT_FFF)_ = the increment of volume received X% of the prescribed dose between VMAT_FF and VMAT_FFF.

The dose gradients in VMAT_FFF treatment techniques were superior to VMAT_FF plans, whether GI_High_ or GI_Low_ were analyzed in Table 1 (*P* < 0.001). Figure 4a, 4b, 4c and 4d showed the linearity of the difference in ΔR_(R50%-R90%)_, ΔR_(R25%-R50%)_, GI_High_ and GI_Low_, respectively, on the diameter of PTV using different planning techniques. For VMAT_FFF plans, the equivalent sphere semidiameter was decreased by approximate 0.01 cm compared to VMAT_FF on the regions of higher doses (from 18 Gy to 10 Gy). In addition, by the use of FFF beams, brain irradiation volumes were reduced by about 2% in low-dose regions (from 10 Gy to 5 Gy) (Figure 4e and Figure 4f).

### MU and beam-on time

For the efficiency analysis, the MU, BOT, gantry speed, treatment delivery time and MDR were listed in Table 2. No statistically significant difference was detected in MU between the two techniques. VMAT_FFF plans provided a significant decrease by 42.8% regarding the mean BOT compared to VMAT_FF. Analyzing the MDR and gantry speed for these two beams, we found an increase of 75.8% and 73.5%, respectively, for VMAT_FFF planning techniques. The mean treatment delivery time in VMAT_FFF plan was 20.63 ± 0.83 minutes, representing an average of 16.1% reduction compared to VMAT_FF plan (Table 2).

**Table 2:** Efficiency analysis for two treatment plans using FF and FFF beams regarding number of monitor units (MU), beam-on time (BOT), treatment delivery time and mean dose rate (MDR)

	VMAT_FF	VMAT_FFF	*P* value
MU	5669 ± 383	5699 ± 430	0.226
BOT (min)	9.53 ± 0.6	5.45± 0.09	<0.001
Treatment delivery time (min)	24.59 ± 0.94	20.63 ± 0.83	<0.001
MDR (MU/min)	595 ± 9	1046 ± 78	<0.001
Gantry speed (deg/s)	3.4 ± 0.2	5.9 ± 0.1	<0.001

*Abbreviations*: VMAT_FF: VMAT plans using conventional flattened (FF) beams; VMAT_FFF: VMAT plans using flattening filter free (FFF) beams.

## DISCUSSION

A comparative appraisal of two different treatment techniques using FF and FFF beams for single brain metastasis was addressed in our study. Dosimetric superiority was shown in VMAT_FFF plans, and a time efficient treatment was also provided in 6X FFF beams which it was a good choice for precision ablative radiation therapy (PART). However, dose distributions achieved with FFF beams are similar or comparable to those with FF beams by a few investigators for brain metastases [15–16]. They only showed that FFF beams potentially enable faster dose delivery substantially shorten treatment delivery time.

Besides the treatment efficiency, the dose gradient is also very important for SRS especially with high single-fraction dose (20 Gy) in brain metastases. For PART, the deeper in dose gradient, the more superior in brain sparing is obtained. To make the dosimetric comparison more conveniently and effectively between FF and FFF beams, firstly, only single brain metastasis and with a maximum diameter smaller than 3 cm were selected in this treatment planning study from our department. Secondly, for each patient, both VMAT_FF and VMAT_FFF plans have followed the same gantry arcs, collimator angles, optimization objectives and priorities, even the timing of priority change are kept the same at best in both optimization processes. A VMAT plan was first generated by Eclipse treatment planning system with FF beams. In the research of dosimetric comparison, the plan quality should be made at best. In this study, the brain is the only one organ at risk, and the dose gradient is greatly important for brain sparing. During the process of planning optimization, five help ring structures were added and two steps were applied to optimize the dose gradient in brain due to that the optimization objectives are contradictory between target volume and normal tissues. Only when an obtuse angle for PTV was formed in DVH (Figure 1c), there was optimized meaningful for the optimization objectives of normal tissues. The obtuse angle of PTV means that the homogeneity will be weakened in target volume. By the use of FFF beams, all of the planning conditions were kept the same to avoid bias, and the data summarized in this report demonstrated that VMAT_FFF plans resulted in superior brain sparing compared to FF beams. Although it does not seem any difference with the naked eye in dose distributions as shown in Figure 2, there are still approximate 2% reductions in low-dose regions (about 5-10 Gy) with FFF beams for brain irradiation (Figure 4e, 4f). These 2% reductions in dose exposure may have clinical significance for the decrease of radiation-induced brain toxicity, especially with 20 Gy in single-fraction dose. In addition, we observed that in homogeneity VMAT_FFF plans were superior to FF plans with a reduction of 3.7% in the mean V_110%_ (*P* < 0.001). The homogeneity is also an important factor that influences the plan quality.

FFF beam is an efficient technique depends on the single-fraction dose > 5 Gy [16], while the dosimetric advantage is due to the non-flat profile and the lower peripheral dose. Compared to traditional beam with flat profile, the integral dose at the same depth for FFF beam is decreased in peripheral field [18]. Some recent treatment planning studys in our department indicated that the healthy tissues would be received less dose exposure with FFF beams in squamous cell carcinoma of the scalp [23] and breast cancer [24] compared to FF beams. In this study, the isocenter of fields in all plans was set to the geometric center of target volume. With respect to percent depth dose (PDD) distribution, both the central axis and off-axis PDD distribution show that FFF beam especially in peripheral field is a softer than traditional beam resulting in less exit dose. Therefore, further brain sparing could be provided by the use of FFF beams, and a negative correlation was found between the percentage reduction in low-dose regions and the diameter of the treated volume (Figure 4e, 4f).

In summary, our results demonstrate that FFF plans show superior quality compare to FF plans with respect to single-fraction stereotactic radiosurgery in single brain metastasis. By the use of FFF beams at high dose rate, VMAT_FFF plans would not only reduce the treatment time, but also have lower volumes in low-dose regions and provide further brain sparing by potentially reducing acute radiation-induced toxicity. The homogeneity and dose gradients in FFF beams were superior to FF beams. For high dose per fraction, the FFF beams are a good choice for precision ablative radiation therapy.

## MATERIALS AND METHODS

### Patients and delineation

68 CT scans of patients with single brain metastasis were involved in this planning study. The diameter of tumor was smaller than 3 cm, and the tumor location was randomly distributed with a minimum distance to brainstem of greater than 2 cm. All patients were scanned by a planning computed tomography in 2 mm slice intervals (General Electric Medical Systems, CT Lightspeed 16). The gross tumor volume (GTV) was delineated by a radiation oncologist. The planning target volume (PTV) was generated from the GTV plus a symmetrical 3 mm margin. Mean PTV volume and standard deviation were 5.35 ± 3.88 cm^3^ (range: 0.3-14.1 cm^3^). With the aim of improving the dose gradient, five additional structures (Ring1, Ring2, Ring3, Ring4 and Ring5) were used during optimization (Figure 1a). The definitions of the ring width were 0.3 cm, 0.4 cm, 1 cm, 1 cm and 1cm, respectively. Organs at risk (OAR) such as brain stem, optic nerves, optic chiasma and lens were outlined in the axial CT sections.

### Treatment planning

Treatment plans were generated for a TrueBeam™ linac (Varian Medical Systems, Palo Alto, CA) equipped with standard Millennium MLC with 120 leaves (0.5 cm spatial resolution at isocenter in the inner 20 cm and 1.0 cm spatial resolution for the 2×10 cm outer length of the field). Every patient was planned with VMAT (RapidArc^®^, Varian Medical Systems) technique using 6X-FF and 6X-FFF beams in the Eclipse^®^ treatment planning system (Varian Medical Systems, PRO 11.0, AAA 11.0). The maximum dose rate was set to 600 MU/min for 6X-FF beams and 1400 MU/min for 6X-FFF beams. The prescribed dose (PD) to the PTV was 1×20 Gy. For comparison purposes, all plans were normalized so that 90% of PTV received 100% of the prescribed dose.

We used one full rotation and 9 partial rotations (10 table angles) for each VMAT plan. For the Eclipse^®^ treatment planning system, the maximum number of arcs we can optimize is 10. For 9 couch rotations, the angles of couch were 18°, 36°, 54°, 72°, 90°, 342°, 324°, 306° and 288°; and the partial arcs were ranged from 179°-320°, 320°-179°, 179°-320°, 320°-179°, 179°-320°,181°-40°, 40°-181°, 181°-40° and 40°-181°. In both VMAT_FF and VMAT_FFF plans for every patient, the same objectives were used and the maximum dose within the PTV receiving 110% of the prescribed dose was allowed. To minimize dose spread outside the PTV, the normal tissue objective automatic tool in Eclipse TPS was used. More importantly, to maximize the dose gradient, five ring structures and corresponding dose constraints were added during optimization (Figure 1b and 1c). In the process of optimization, two steps were applied to optimize the dose gradient in brain. In the first stage, the optimized objectives were biased towards PTV to form an approximate right angle in dose-volume histogram (DVH) (Figure 1b). In the second stage, we were biased towards the objectives for five ring structures and an obtuse angle for PTV was formed in DVH (Figure 1c).

### Plan comparisons and evaluation tools

Dosimetric evaluation was performed according to the standard DVH. For PTV coverage, the mean dose (D_mean_) and the maximum dose (D_max_), the values of D_2%_ (dose received by 2% of the PTV) and V_110%_ (volume of the PTV receiving at least 110% of the prescribed dose) were compared between FF and FFF beams. The homogeneity of the PTV was evaluated with a homogeneity index (HI), which was defined as: HI = D_5%_/D_95%_ (dose received by 5%, and 95% of the PTV). The conformity of the PTV was measured with a conformity index (CI), which was defined as: CI = (V_PTV_/TV_PV_)/(TV_PV_/V_TV_) [21]. V_PTV_ is the volume of PTV. TV_PV_ is the portion of the V_PTV_ within the 90% of prescribed isodose line. V_TV_ is the volume of the body that received 90% of the prescribed dose.

The dose gradient of the plans was expressed in terms of the gradient index (GI) [22]. For the regions of higher doses and lower doses, the GI_High_ (V_50%Presc.Dose_/V_90%Prescr.Dose_) and GI_Low_ (V_25%Presc.Dose_/V_50%Prescr.Dose_) were introduced in this paper, respectively. V_X%Presc.Dose_ is the volume of the body that received X% of the prescribed dose. In addition, to describe the dose gradient more intuitively and provide reference for radiation oncologists, the values of ΔR_(R50%-R90%)_, ΔR_(R25%-R50%)_, ΔV_50%(VMAT_FF-VMAT_FFF)_ and ΔV_25%(VMAT_FF-VMAT_FFF)_ were analyzed. ΔR_(R50%-R90%)_ is the distance from 90% isodose line to 50% isodose line. (R_X(%)_ is equivalent sphere semidiameter for the volume received X% of the prescribed dose). ΔR_(R25%-R50%)_ is the distance from 25% isodose line to 50% isodose line. ΔV_X%(VMAT_FF-VMAT_FFF)_ is the delta value of volume received X% of the prescribed dose between VMAT_FF and VMAT_FFF plan.

For organs at risk (OAR) irradiation, such as brain stem, optic nerves, optic chiasma and lens, there were no clinically meaningful because the distance from the PTV to OARs was too large in all patients. Total monitor units (MU), beam-on time (BOT), gantry speed and mean dose rate (MDR) were compared. Statistical analyses were performed in order to compare the different irradiation techniques using 6X-FF or 6X-FFF beams. Relative dosimetric changes were compared using the paired, two-tailed Wilcoxon signed-rank test. *P* ≤ 0.05 was considered statistically significant.
